# Supplementary material for: Sharp Wave-Ripples in Human Amygdala and Their Coordination with Hippocampus during NREM Sleep
Source: Cereb Cortex Commun. 2020 Aug 20;1(1):tgaa051. doi: 10.1093/texcom/tgaa051 (PMC7521160; doi:10.1093/texcom/tgaa051)
Supplement: supplementaryMaterial_tgaa051 [file supplementarymaterial_tgaa051.pdf]

Supplementary Table

	sex	age (y)	duration of epilepsy (y)	diagnosis	recording side	anti-convulsant	anti-depressant
p1	F	31	18	medial temporal lobe epilepsy, no structural correlate, right-dominant seizure focus	L	oxcarbazepine[1]	-
p2	F	23	10	medial temporal lobe epilepsy, hippocampal sclerosis left	R	lamotrigine[2]	sertraline[3]
p3	M	30	20	focal epilepsy, heterotopia left temporo-occipital	R	lamotrigine[2]<br>oxcarbazepine[1]	-
p4	F	23	13	medial temporal lobe epilepsy, hippocampal sclerosis left	R	levetiracetam[4]	-

Supplementary Table 1. Patient demographics, clinical status, and drug regime. For all drugs, plasma concentrations were within the therapeutic range at the moment of recording. [1]Effect on human sleep unknown, but increases NREM and REM sleep in rats (Ayala-Guerrero et al. 2009). [2]Increases REM sleep and N2, decreases N3 (in epilepsy) (Jain and Glauser 2014). [3]Increases sleep latency and delta activity in first sleep cycle (in depression) (Jindal et al. 2003). [4]Increases N2, decreases N3 (in epilepsy) (Jain and Glauser 2014).

Table References

Ayala-Guerrero F, Mexicano G, González V, Hernandez M. 2009. Effect of oxcarbazepine on sleep architecture. Epilepsy & Behavior. 15:287–290.

Jain SV, Glauser TA. 2014. Effects of epilepsy treatments on sleep architecture and daytime sleepiness: An evidence-based review of objective sleep metrics. Epilepsia. 55:26–37.

Jindal RD, Friedman ES, Berman SR, Fasiczka AL, Howland RH, Thase ME. 2003. Effects of Sertraline on Sleep Architecture in Patients With Depression: Journal of Clinical Psychopharmacology. 23:540–548.

Supplementary Figures

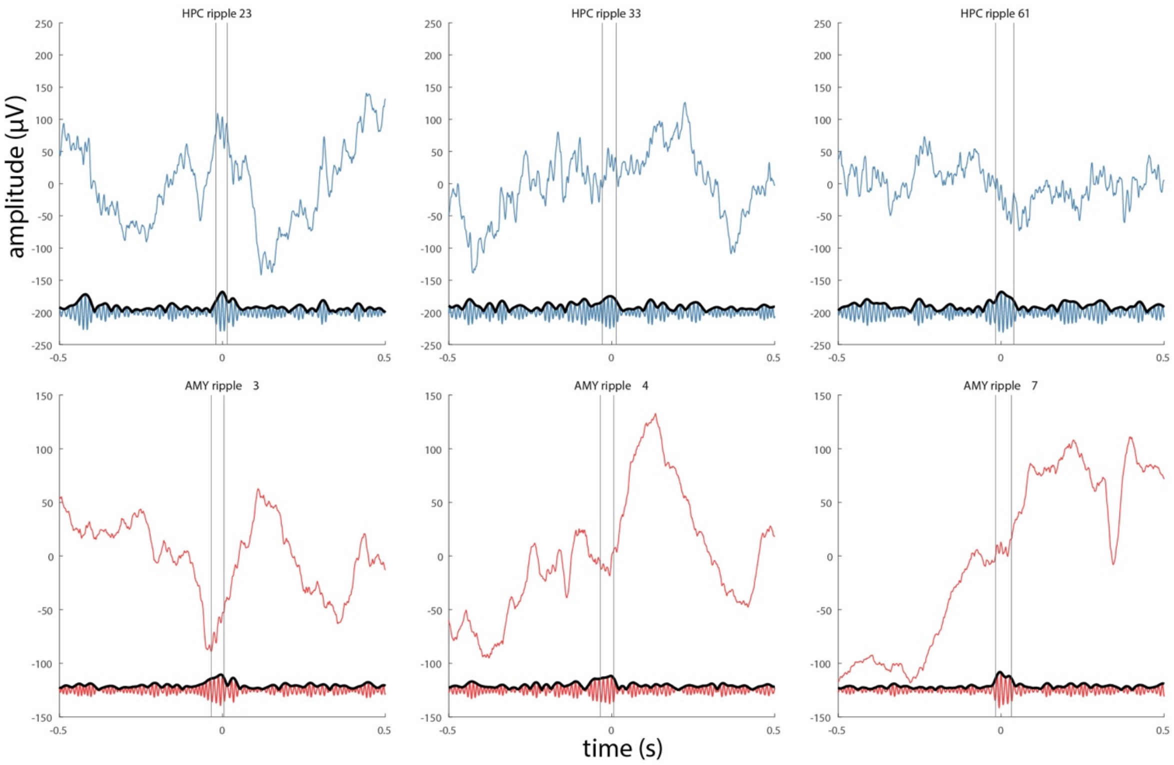

Supplementary Figure 1. Examples of detected ripples for patient p2. Figure layout as in Figure 4.

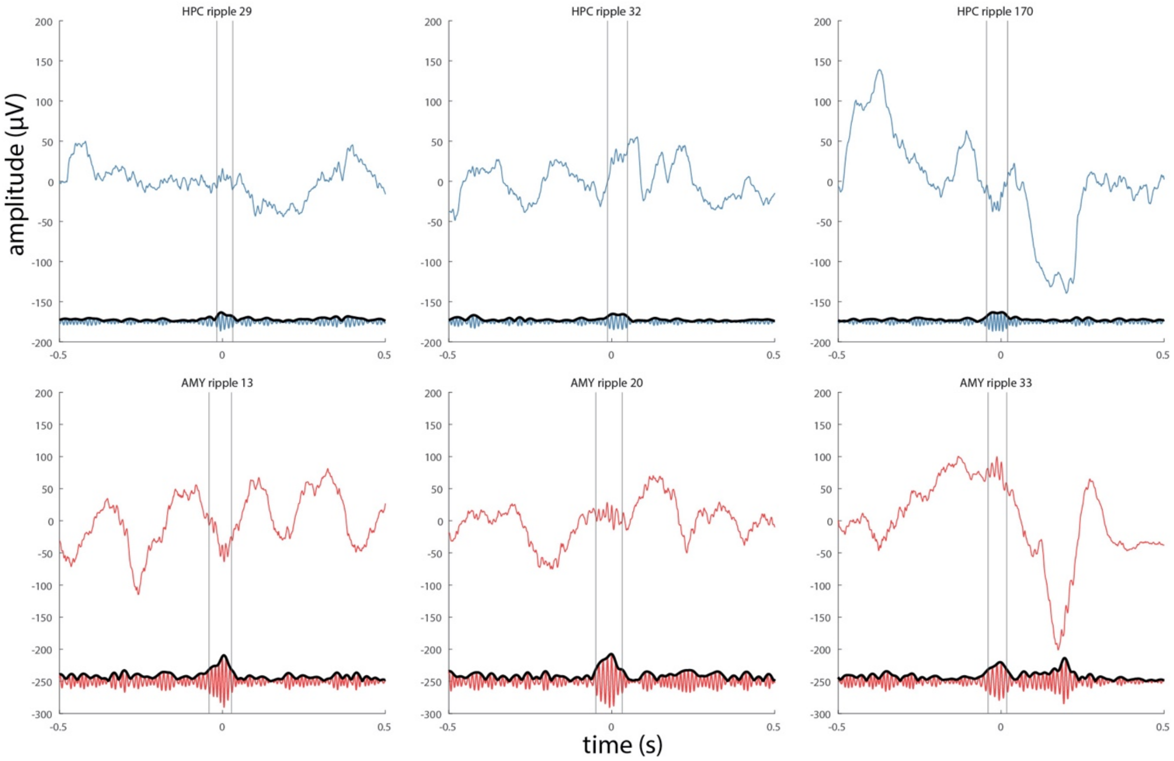

Supplementary Figure 2. Examples of detected ripples for patient p3. Figure layout as in Figure 4.

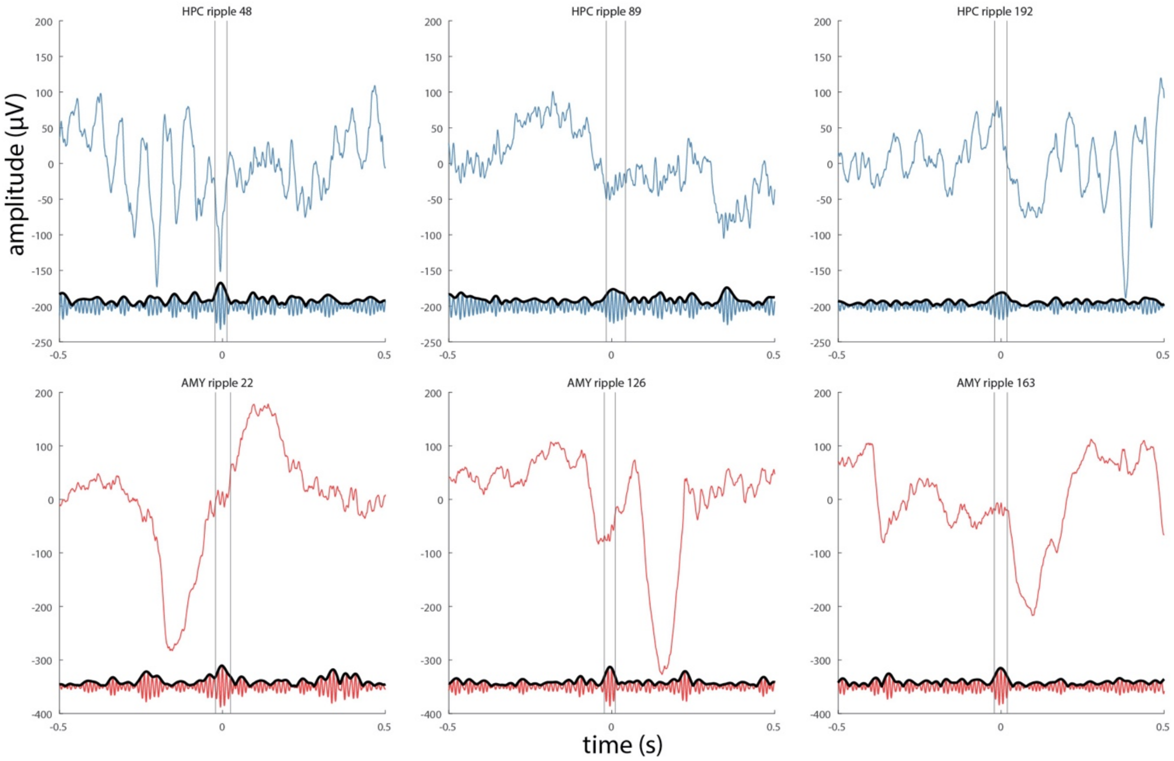

Supplementary Figure 3. Examples of detected ripples for patient p4. Figure layout as in Figure 4. Note spindle rhythmicity in HPC (top-left and top-right panels).

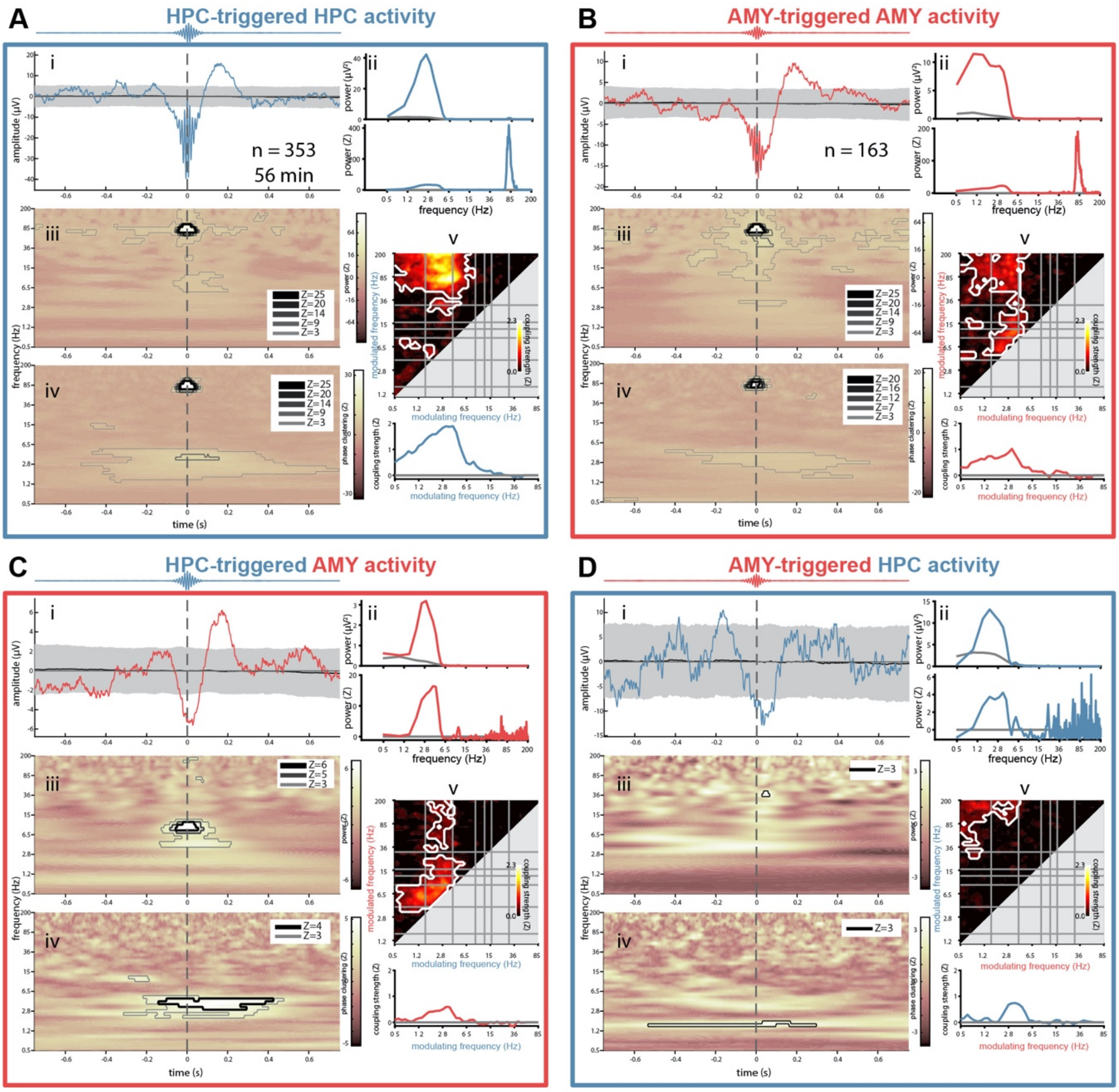

Supplementary Figure 4. Local and interregional ripple-related dynamics in hippocampus and amygdala for patient p1. Panel layout identical to that of Figures 5 and 6.

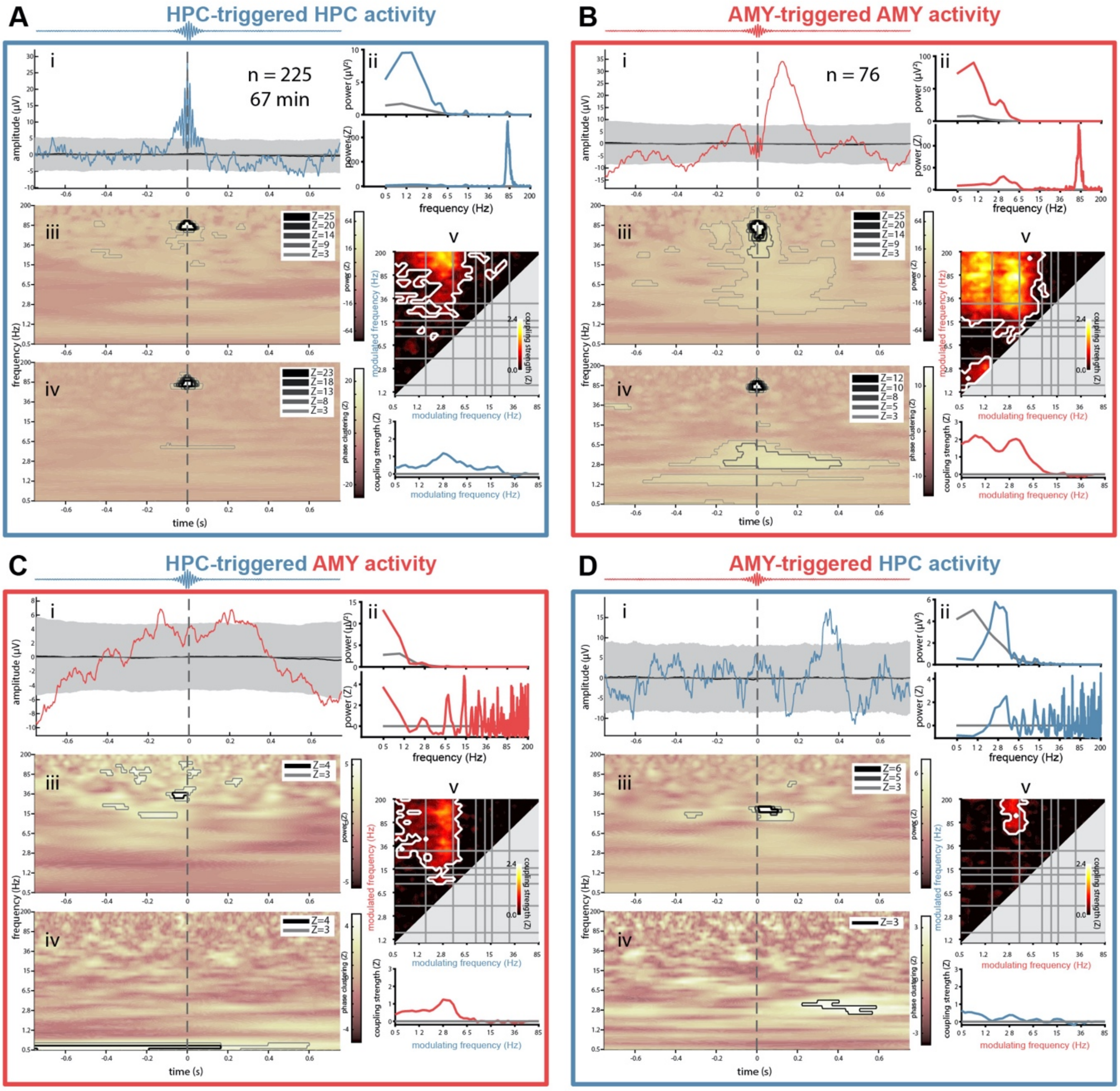

Supplementary Figure 5. Local and interregional ripple-related dynamics in hippocampus and amygdala for patient p2. Panel layout identical to that of Figures 5 and 6.

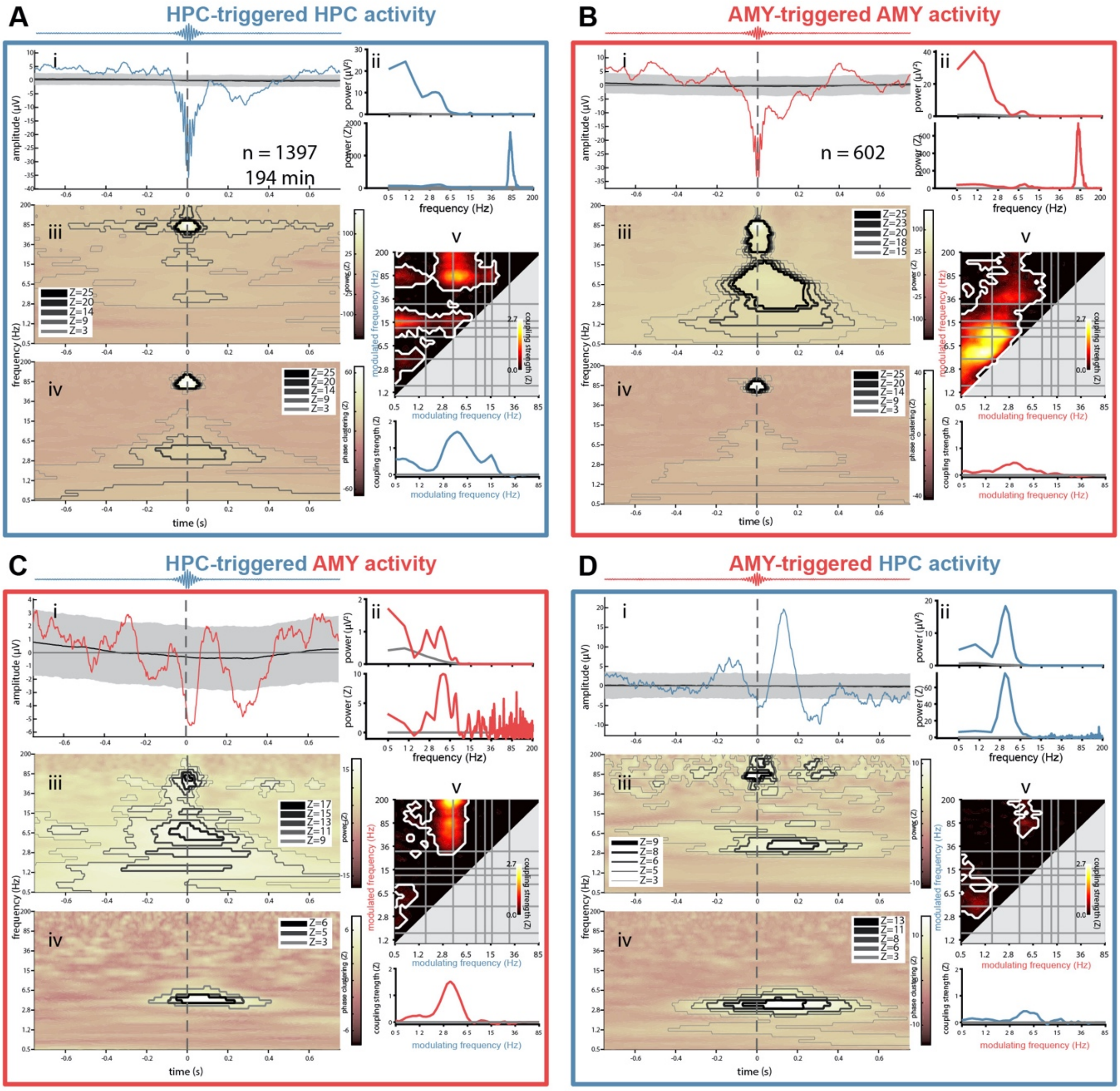

Supplementary Figure 6. Local and interregional ripple-related dynamics in hippocampus and amygdala for patient p4. Panel layout identical to that of Figures 5 and 6.
